# Acute aortic dissection during minimally invasive cardiac surgery: a case report

**DOI:** 10.1186/s40981-025-00771-2

**Published:** 2025-01-31

**Authors:** Taisuke Kumamoto

**Affiliations:** Department of Anesthesiology, Saiseikai Kumamoto Hospital, 5-3-1 Minami-Ku, Chikami Kumamoto, 861-4193 Japan

**Keywords:** Minimally invasive cardiac surgery, Acute aortic dissection, Transesophageal echocardiography, Cardiopulmonary bypass, Retrograde perfusion

## Abstract

**Background:**

Management of acute aortic dissection (AAD) caused by retrograde perfusion through the femoral artery during minimally invasive cardiac surgery (MICS) remains controversial. We present a case of AAD occurring during the late cardiopulmonary bypass (CPB) phase, which was successfully managed by vascular graft replacement, without altering the blood supply route.

**Case presentation:**

A 63-year-old man was scheduled for totally endoscopic aortic valve replacement. CPB was initiated through the right femoral artery and venous cannulation. Approximately 120 min after the initiation of CPB, mean arterial pressure and bilateral cerebral regional oxygen saturation temporarily decreased. Transesophageal echocardiography revealed type A AAD. Cerebral perfusion was preserved, allowing us to proceed to deep hypothermic circulatory arrest and successfully perform ascending aortic replacement without altering the blood supply route.

**Conclusions:**

In MICS, continuous monitoring is crucial as AAD can occur at any point during CPB, and early detection enables successful outcomes.

## Background

In minimally invasive cardiac surgery (MICS) performed via right thoracotomy, the arterial cannula for cardiopulmonary bypass (CPB) is typically inserted through the femoral artery (FA). However, retrograde perfusion can result in complications, such as acute aortic dissection (AAD) [1].

AAD during MICS is rare, and its management is not well established. AAD most often occurs immediately after the initiation of CPB [1, 2]. In such cases, discontinuation of CPB and perfusion of the true lumen by the native heart are generally recommended [1, 3]. However, if AAD occurs during the later stages of CPB, when discontinuing CPB is not feasible, there is no consensus on the optimal management [4]. Potential approaches include securing an alternative blood supply, reducing the flow of the CPB pump, or initiating systemic cooling to protect vital organs [1].

Here, we report a rare case of AAD occurring during the late CPB phase in MICS, which was successfully managed by replacement of the vascular graft without altering the blood supply route.

## Case presentation

We obtained written informed consent from the patient for publication of this case report.

A 63-year-old man (height: 165 cm, weight: 73 kg) with a medical history of hypertension presented with exertional dyspnea. Transthoracic echocardiography revealed severe bicuspid aortic stenosis, while computed tomographic angiography did not show significant calcification or atherosclerosis in the aorta. The diameter of the right FA was 10.5 mm. The patient was scheduled to undergo totally endoscopic aortic valve replacement (AVR).

Upon the patient’s arrival in the operating room, the left radial artery was cannulated to monitor his arterial blood pressure. Following the induction of general anesthesia, a transesophageal echocardiography (TEE) probe was inserted. To assess cerebral perfusion, the patient’s regional oxygen saturation (rSO_2_) was monitored bilaterally on his forehead. The patient was positioned in a left semi-lateral position, with his right upper limb elevated and placed in front of his face.

**Fig. 1:**
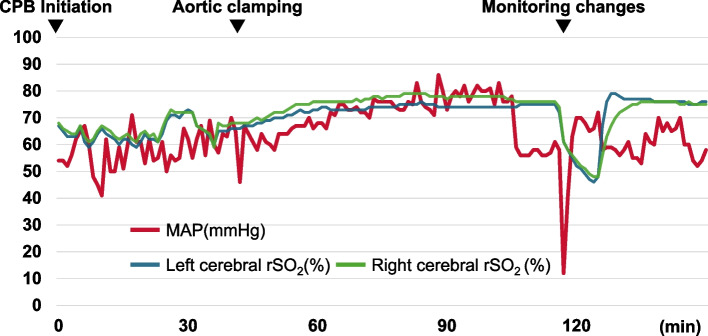
Mean arterial pressure and cerebral regional oxygen saturation during cardiopulmonary bypass. Approximately 120 min after the initiation of cardiopulmonary bypass (CPB), mean arterial pressure (MAP) and bilateral cerebral regional oxygen saturation (rSO_2_) temporarily decreased

Following a right fourth intercostal thoracotomy, the FA was exposed through a right groin incision. TEE and X-ray fluoroscopic guidance were used to insert a 25-Fr venous cannula through the right femoral vein into the right atrium and a 19-Fr arterial cannula through the right FA. Peripheral vascular cannulation was successfully performed on the first attempt without difficulty. After the initiation of CPB, the pericardium was incised to expose the ascending aorta. After cannulation, initiation of CPB, and aortic clamping, TEE confirmed the absence of AAD. The temperature of the urinary bladder was cooled to 32 °C during the excision of the calcified bicuspid valve and annular debridement. The flow of the CPB pump was targeted at 2.6 L/min/m^2^, with the pressure of the CPB line maintained between 250 and 260 mm Hg. The mean arterial pressure (MAP) was maintained between 60 and 80 mm Hg. The bilateral forehead rSO_2_ values ranged from 60 to 80% (Fig. 1).

**Fig. 2:**
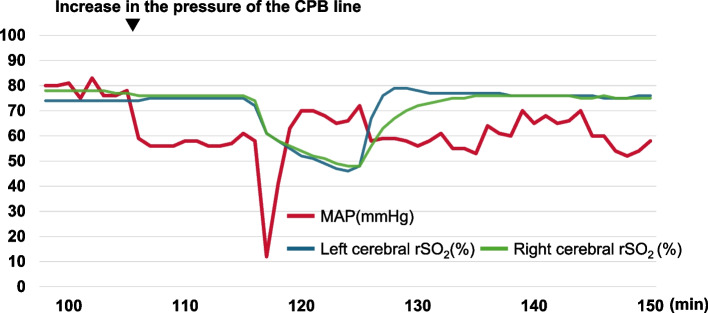
Mean arterial pressure and cerebral regional oxygen saturation between 100 and 150 min during cardiopulmonary bypass. Mean arterial pressure (MAP) abruptly decreased to 12 mmHg and returned to baseline at 117 min after the initiation of cardiopulmonary bypass (CPB). Bilateral cerebral regional oxygen saturation (rSO_2_) gradually decreased to 46% over 8 min and then returned to the baseline in the subsequent 2 min

**Fig. 3:**
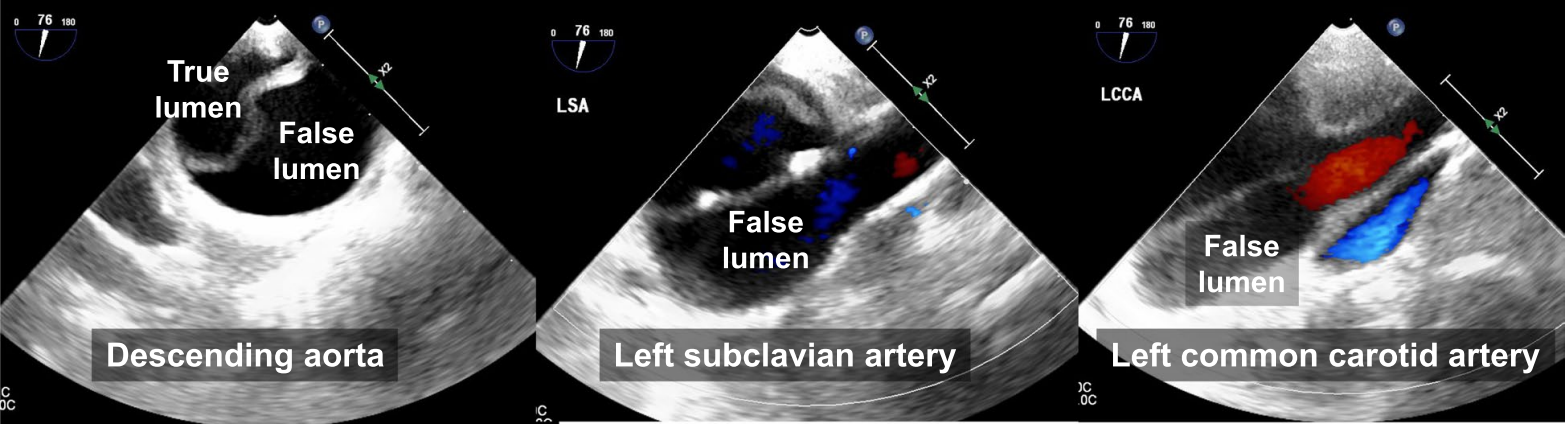
Intraoperative transesophageal Doppler echocardiography showing acute aortic dissection in the descending aorta (left), left subclavian artery (middle), and left common carotid artery (right)

Approximately 107 min after the initiation of CPB, the pressure of the CPB line gradually increased to 285–295 mm Hg and was closely monitored. At 117 min, while the mechanical valve was being sutured to the annulus, the blood pressure waveform of the left radial artery abruptly disappeared, and the MAP decreased to 12 mm Hg and then returned to baseline within 30 s (Fig. 2). Around the same time, the rSO_2_ values measured bilaterally at the forehead gradually decreased to 46% over 8 min, before returning to baseline within the subsequent 2 min (Fig. 2). TEE revealed an AAD extending from the descending aorta to the left common carotid artery (Fig. 3); the ascending aorta could not be examined. Although the ascending aorta distal to the cross-clamp site could not be visualized on the video monitor, carotid ultrasound revealed that the AAD had extended to the right common carotid artery. The flow of the CPB pump was reduced to 1.8 L/min/m^2^, and the temperature of the urinary bladder was cooled to 24.6 °C. We decided to perform aortic replacement after completing the endoscopic AVR without altering the route of the blood supply, since the MAP and rSO_2_ values were stable.

After the mechanical valve was sutured to the annulus, a median sternotomy was performed, which revealed a dilated and discolored aortic arch. During deep hypothermic circulatory arrest (DHCA), a branched vascular graft was anastomosed to the ascending aorta, and the patient was weaned off the CPB pump without difficulty. The durations of the operation, CPB, and aortic cross-clamping were 575, 376, and 253 min, respectively. The patient’s postoperative course was uneventful, and no complications were noted. Postoperative computed tomography revealed intimal tears located above the common iliac bifurcation and within the left subclavian artery.

## Discussion

We identified two critical issues related to AAD occurring during this MICS. First, AAD may occur long after retrograde perfusion through the FA has been initiated. Second, even after AAD had occurred in association with retrograde perfusion, it may still be possible to retain the existing route for blood supply.

Although AAD most commonly occurs at the onset of CPB or early in the procedure [1, 2], it can also occur long after retrograde perfusion through the FA has been initiated. It has been reported that 26% of AAD cases occur during the midportion of CPB [1]; therefore, anesthesiologists must remain highly vigilant for any changes in monitoring during this period. When AAD is attributed to peripheral cannulation, it may result from direct trauma caused by the cannula or the blood flow jet [5]. In our patient, the tip of the arterial cannula may have shifted just before the line pressure of the CPB increased, potentially exerting external force on the aortic intima. However, since the intimal tear was located above the bifurcation of the common iliac artery, we hypothesized that the prolonged exposure of the aortic wall to the blood flow jet contributed to the event. It was considered that intimal tear caused by the blood flow jet led to the progression of AAD, but the formation of a reentry within the left subclavian artery allowed the decreased MAP and rSO_2_ to return to baseline. A small arterial cannula is known to generate high shear stress on the aortic wall, and in MICS, the arterial cannula inserted through the FA is smaller in diameter than the one used for the ascending aorta, leading to higher pressure in the CPB line and increased stress on the aortic wall [6]. Since the flow of the CPB pump is positively correlated with the degree of vascular injury [7], reducing the CPB pump flow should be considered. Based on these considerations, our MICS protocol was updated as follows: the body temperature was lowered to 30 °C during prolonged CPB, and the target CPB pump flow was adjusted from 2.6 to 2.2 L/min/m^2^ to minimize shear stress on the aortic wall.

When an arterial cannula is inserted through the ascending aorta via a median sternotomy, the early detection of AAD through signs such as aortic enlargement and discoloration should facilitate the prevention of FA dissection, with the FA often serving as an alternative blood supply route [1, 2]. In contrast, when the arterial cannula is inserted through the FA, the early detection of AAD is more difficult. Major progression of AAD has often already occurred by the time it is identified [1]. Therefore, the management of AAD with retrograde extension via cannulation of the FA remains controversial.

Maintaining cerebral perfusion is essential for preventing a poor outcome after the occurrence of iatrogenic AAD by transitioning promptly to DHCA and minimizing the duration of DHCA and CPB [1, 8]. In our patient, AAD had extended into the systemic vessels, and the patient’s surgical position, with his right upper limb elevated, made changing the route of the blood supply challenging. We thought that changing the route of the blood supply route would prolong the time required to achieve DHCA and extend the time on the CPB. Furthermore, the values of MAP and rSO_2_ remained stable, and cerebral perfusion was confirmed by carotid ultrasound; therefore, the route of the blood supply route was not changed.

After the diagnosis of AAD, the priority was to reduce the flow of the CPB pump and transition to DHCA as quickly as possible. Considering that the performance of a total arch replacement would prolong the durations of DHCA and CPB, an ascending aortic replacement was performed instead. Moreover, the MICS-AVR was successfully completed, while preparations for emergency median sternotomy were underway. However, if manipulation of the aortic valve requires additional time, AVR can also be performed during the rewarming phase following replacement of the vascular graft [9].

The early detection of retrograde dissection during MICS and the prevention of its progression to type A AAD are crucial. However, by the time changes in MAP or rSO₂ indicate its presence, the AAD has often already advanced significantly. Carefully monitoring changes in the pressure of the CPB line and promptly performing TEE when an increase in line pressure is observed are essential for facilitating the early detection of AAD.

In conclusion, in MICS, AAD can occur at any point during CPB, so continuous careful monitoring is essential, and early detection may allow the performance of an ascending aortic replacement without altering the blood supply route.

## Data Availability

The datasets are available from the corresponding author on reasonable request.

## Abbreviations

MICS: Minimally invasive cardiac surgery
CPB: Cardiopulmonary bypass
FA: Femoral artery
AAD: Acute aortic dissection
AVR: Aortic valve replacement
TEE: Transesophageal echocardiography
rSO_2_: Regional oxygen saturation
MAP: Mean arterial pressure
DHCA: Deep hypothermic circulatory arrest
